# Optimization of Ultrasound-Assisted Extraction of (−)-Stepholidine from *Onychopetalum amazonicum* Leaves Using Response Surface Methodology

**DOI:** 10.1021/acsomega.5c06822

**Published:** 2025-11-11

**Authors:** Bruna Ribeiro de Lima, Kidney de Oliveira Gomes Neves, Lucas Apolinário Chibli, Ana Paula Alfaia Castro, Rebeca dos Santos França, Giovana Anceski Bataglion, Marcos Batista Machado, Afonso Duarte Leão de Souza, Hector Henrique Ferreira Koolen, Maria Lúcia Belém Pinheiro, Felipe Moura Araújo da Silva

**Affiliations:** † Departamento de Química, 67892 Universidade Federal do Amazonas (UFAM), 69080-900 Manaus, Amazonas, Brazil; ‡ Centro de Apoio Multidisciplinar, Universidade Federal do Amazonas (UFAM), 69080-900 Manaus, Amazonas, Brazil; § Departamento de Pesquisa e Desenvolvimento de Extratos Botânicos e Desidratados, Duas Rodas, 89251-901 Jaraguá do Sul, Santa Caterina Brazil; ∥ Escola Superior de Ciências da Saúde, Universidade do Estado do Amazonas, (UEA), 690065-130 Manaus, Amazonas, Brazil; ⊥ Coordenação de Tecnologia e Inovação (COTEI), 191073 Instituto Nacional de Pesquisas da Amazônia, 69067-375 Manaus, Amazonas, Brazil

## Abstract

(−)-Stepholidine, a naturally occurring alkaloid found in the leaves of Onychopetalum amazonicum (Annonaceae), has shown significant pharmacological potential and serves as an important precursor in the synthesis of bioactive compounds. Currently, ultrasound-assisted extraction (UAE), combined with optimization through response surface methodology (RSM), has emerged as a promising strategy to enhance the efficiency of extraction processes. This study aimed to develop an efficient method for extracting (−)-stepholidine from the leaves of *O. amazonicum*. Powdered leaves were extracted using methanol-based systems assisted by ultrasound, following a central composite rotatable design (CCRD) with three factors: plant-to-solvent ratio (X_1_), methanol concentration (X_2_), and extraction time (X_3_). The samples were quantified by NMR using the PULCON method. The model was validated by ANOVA, and the optimal conditions were determined using RSM. The factors X_2_^2^ (methanol percentage), X_3_ (extraction time), and the interaction X_1_·X_3_ (plant-to-solvent ratio × extraction time) significantly influenced (−)-stepholidine concentration (*p* < 0.05). The model showed a good fit (R^2^ = 0.728) and was statistically significant (*p* = 0.0005). The optimal conditions identified were as follows: a plant-to-solvent ratio of 1:10, 100% methanol, and 20 min of extraction. Experimental validation yielded an average (−)-stepholidine concentration of 82.8 ± 1.3 mg per g of extract, close to the predicted value of 81.2 mg/g. This study demonstrates that optimizing extraction parameters, such as the plant-to-solvent ratio, methanol percentage, and extraction time, is crucial for maximizing (−)-stepholidine recovery. These findings also support the potential of UAE combined with qNMR as a reliable and reproducible approach for the extraction and quantification of bioactive compounds from plant matrices.

## Graphical abstract

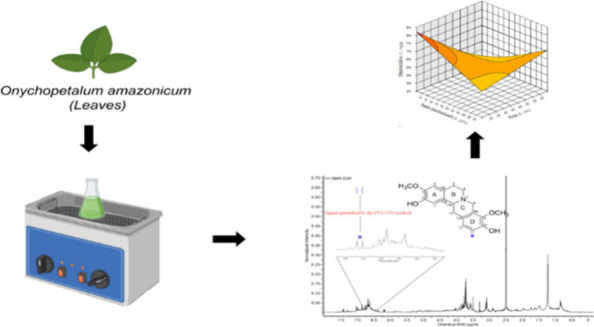

## Introduction

(−)-Stepholidine, an isoquinoline-derived alkaloid that is abundantly found in the leaves of *Onychopetalum amazonicum* (Annonaceae),^1^ has attracted the attention of researchers worldwide due to its increasingly recognized list of biological activities. Its pharmacological effects are particularly notable within the central nervous system (CNS). The primary pharmacological action of (−)-stepholidine is its the modulation of dopaminergic receptors, acting as a partial agonist at D1 receptors and as an antagonist at D2 receptors. This dual action makes it potentially useful in the treatment of disorders such as schizophrenia, with fewer side effects compared to traditional antipsychotics.^2–6^

In the context of neurodegenerative diseases, such as Alzheimer’s, (−)-stepholidine has been shown to reverse memory deficits in animal models through the dopaminergic pathway.^7^ *In vitro* studies have also demonstrated its inhibition of acetylcholinesterase (AChE) activity.^8^ Additionally, (−)-stepholidine protects neurons against damage induced by neurotoxic substances such as methamphetamine (METH),^9^ and reduces the self-administration of drugs like heroin and METH by acting on the brain’s reward system.^10,11^ Another important effect is its ability to reduce morphine-induced drug-seeking behavior, even after such associations have already been established.^12^ Beyond its pharmacological properties, (−)-stepholidine has emerged as an important starting material in the synthesis and semisynthesis of bioactive compounds, whose derivatives display significant pharmacological activity within the CNS.^8,13,14^

To support such applications and advance pharmacological research, the development of efficient extraction methods for plant matrices is crucial. Traditionally, maceration has been the most commonly used method for extracting (−)-stepholidine, although cold percolation has also been reported.^15–19^ However, both methods have limitations, including long extraction times, high solvent consumption, and lower efficiency in releasing bioactive compounds.^20,21^ In view of these limitations, ultrasound-assisted extraction (UAE) has emerged as a promising alternative. UAE uses ultrasonic waves to disrupt plant cells, thereby facilitating the effective release of bioactive compounds.^22^ Compared to traditional methods, UAE offers several advantages, such as reduced extraction times, lower solvent consumption, higher extraction yields, and operation at lower temperatures, thereby minimizing thermal degradation of sensitive compounds.^23,24^

The optimization of the extraction process is crucial for maximizing both the yield and quality of the extract. The Design of Experiments (DoE) provides a structured framework for simultaneously evaluating multiple variables, such as plant-to-solvent ratio, solvent concentration, extraction time, and temperature.^25^ Within this framework, Response Surface Methodology (RSM) is a powerful technique used to model and optimize responses by exploring the relationships between these factors. Compared to one-factor-at-a-time (OFAT) and trial-and-error methods, RSM significantly reduces material consumption and the time required for optimization. It is a widely used approach for modeling and optimizing the extraction of bioactive compounds.^26,27^ The Central Composite Rotatable Design (CCRD) is a specific type of experimental design commonly employed in RSM, which allows for the estimation of linear, interaction, and quadratic effects of the variables. Together, these approaches enable the development of mathematical models that systematically identify optimal extraction conditions by considering variable interactions, offering a more efficient alternative to traditional methods.^25,28,29^

In addition to optimizing the extraction process, the choice of an analytical technique for determining bioactive compounds is fundamental to ensuring the quality and reproducibility of results. In this context, nuclear magnetic resonance (NMR) spectroscopy, an absolute technique that is combined with the PULCON method (*Pulse Length-Based Concentration Determination*), stands out as a powerful tool. This approach enables the quantification of compounds in complex mixtures without the need for specific standards, based on the principle of reciprocity, which correlates absolute intensities in one-dimensional (1D) NMR spectra (qNMR).^30,31^ The PULCON method has been applied to the analysis of plant extracts, offering significant advantages, as it eliminates the need for isolation and purification of standards, simplifying the analytical process and ensuring greater precision in the quantification of bioactive compounds.^32–34^

Therefore, this study aims to integrate advanced extraction techniques, such as UAE, with systematic optimization methods (DoE and RSM), as well as quantification by nuclear magnetic resonance (qNMR), in order to develop an efficient method for extracting (−)-stepholidine from the leaves of *O. amazonicum*.

## Results and Discussion

### Quantitative NMR Analysis, Experimental Design, and Statistical Modeling

Prior to quantification, ^1^H NMR (Figure 1), along with HSQC and HMBC experiments (Supporting Information, Figures S1–S4), were performed to confirm the presence of characteristic (−)-stepholidine signals^1^ in the samples and to verify the absence of signal overlap. Four diagnostic signals were identified in the extracts at δ_H_ 6.76 (H-11), 6.70 (H-12), 6.68 (H-1), and 6.63 (H-4). These correlated with carbons at δ_C_ 116.3 (C-11), 124.3 (C-12), 113.1 (C-1), and 112.6 (C-4), respectively. The HMBC spectrum revealed long-range correlations with key carbons. For instance, δ_H_ 6.76 (H-11) correlated with δ_C_ 126.2 (C-12a) and 146.0 (C-9); δ_H_ 6.63 (H-4) correlated with δ_C_ 29.2 (C-5); δ_H_ 6.68 (H-1) correlated with δ_C_ 59.2 (C-13a); and δ_H_ 6.70 (H-12) correlated with δ_C_ 36.3 (C-13). These data confirmed the presence of (−)-stepholidine in the samples. Among the four signals, only the signal at δ_H_ 6.76 was free from overlap and therefore selected for quantification (Figure 1). Using this signal and the PULCON method, the efficiency of the 17 experimental combinations in recovering (−)-stepholidine was evaluated. The (−)-stepholidine yields ranged from 56.5 ± 0.9 to 76.5 ± 2.3 mg/g.

**Figure 1:**
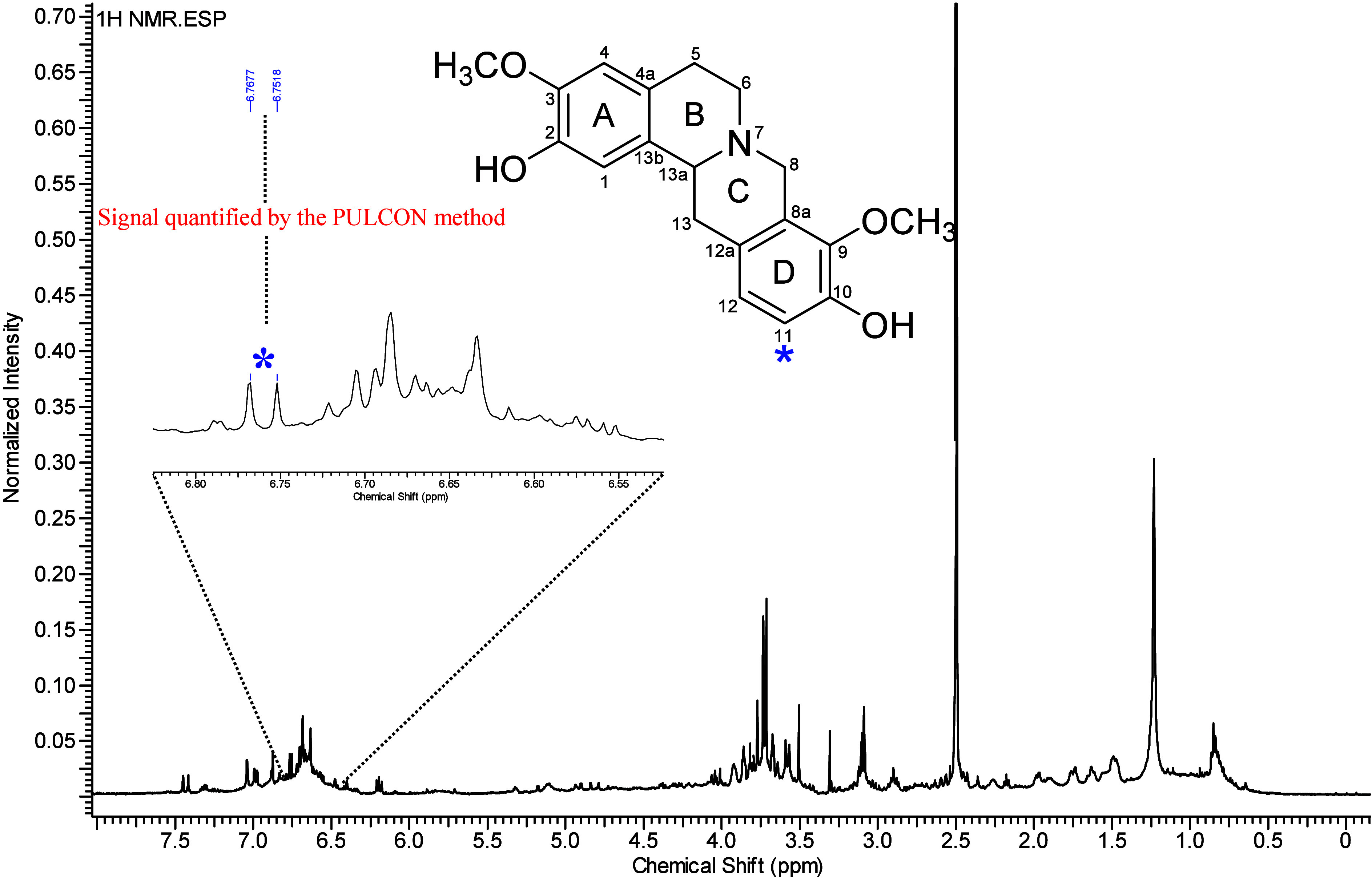
^1^H NMR spectrum of the methanol extract of *O. amazonicum* leaves (DMSO-*d*_6_, 500 MHz), highlighting the signal at 6.76 ppm used for quantification by the PULCON method.

The results of the experimental design using a CCRD with three factors are presented in Table 1. The plant-to-solvent ratio (9.91–30.09 g/mL) was chosen to provide adequate variation in the density of plant material relative to the solvent volume. Excessively high ratios may result in an increased solvent volume, thereby increasing the time required for extract concentration.^21^ The methanol concentration (37.28–100%) was selected based on previous studies of the extraction efficiency of isoquinoline-derived alkaloids reported by Rocha et al.^35^ The extraction time (22.36–60.04 min) was determined taking into account that modern methods, such as ultrasound-assisted extraction (UAE), allow shorter extraction periods compared to traditional techniques, especially for bioactive compounds.^36^ This range is also consistent with that used in studies on alkaloid extraction by UAE.^37^

**Table 1:** CCRD Applied to Optimize (−)-Stepholidine Content in the Extract of *Onychopetalum amazonicum* Leaves

Run	Plant-Solvent (X_1_, g/mL)	Methanol (X_2_, %)	Time (X_3_, min)	(−)-Stepholidine (Y_1_, mg/g^a^)
1	–1 (1:14)	–1 (50)	–1 (30)	76.5 ± 2.3
2	1 (1:26)	–1 (50)	–1 (30)	69.3 ± 0.6
3	–1 –1 (1:14)	1 (87.30)	–1 (30)	74.5 ± 2.7
4	1 (1:26)	1 (87.30)	–1 (30)	71.2 ± 2.0
5	–1 (1:14)	–1 (50)	1 (52.40)	63.4 ± 1.8
6	1 (1:26)	–1 (50)	1 (52.40)	65.5 ± 2.2
7	(1:14)	1 (87.30)	1 (52.40)	56.5 ± 0.9
8	1 (1:26)	1 (87.30)	1 (52.40)	66.2 ± 2.4
9	–1.68 (1:9.91)	0 (68.65)	0 (41.20)	65.7 ± 1.4
10	1.68 (1:30.09)	0 (68.65)	0 (41.20)	68.2 ± 1.9
11	0 (1:20)	–1.68 (37.28)	0 (41.20)	71.8 ± 1.8
12	0 (1:20)	1.68 (100.02)	0 (41.20)	73.8 ± 3.5
13	0 (1:20)	0 (68.65)	–1.68 (22.36)	65.2 ± 1.8
14	0 (1:20)	0 (68.65)	1.68 (60.04)	63.2 ± 1.4
15	0 (1:20)	0 (68.65)	0 (41.20)	65.5 ± 2.3
16	0 (1:20)	0 (68.65)	0 (41.20)	64.8 ± 2.4
17	0 (1:20)	0 (68.65)	0 (41.20)	66.3 ± 2.8

a mg stepholidine per g extract (dry basis).

At a 95% confidence level, the factors X_2_^2^, X_3_, and X_1_·X_3_ showed a significant influence on (−)-stepholidine concentration (*p* < 0.05) (Table 2). The final mathematical model describing this relationship (eq 1) was defined after removing nonsignificant coefficients and recalculating the parameters.

1 $$Y=65.46+2.55X_{2}^{2}-3.17X_{3}+2.79X_{1}\cdot X_{3}$$

**Table 2:** Regression Model Obtained from the CCRD for the Extraction of (−)-Stepholidine from *O. amazonicum*

Name	Coefficient	Standard Error	Calculated -t	p-value
Mean	65.46	0.94	69.65	0.0000
X_2_^2^	2.55	0.79	3.22	0.0067
X_3_	–3.17	0.77	–4.11	0.0012
X_1_·X_3_	2.79	1.01	2.76	0.0162

Considering the maximization of (−)-stepholidine extraction, three main effects were observed. First, the positive quadratic effect of methanol concentration (X_2_^2^) indicates that higher concentrations lead to greater extraction efficiency. Second, the negative effect of extraction time (X_3_) suggests that shorter processes are more efficient. Finally, the positive interaction between the plant-to-solvent ratio (X_1_) and extraction time (X_3_) demonstrates a synergistic effect that enhances extraction efficiency.

The positive effect of higher methanol content on (−)-stepholidine extraction is evident when comparing experiments 2 and 4 in the CCRD. The optimization model also indicated that higher methanol concentrations enhanced extraction, reinforcing this observation. This behavior reflects the strong affinity of (−)-stepholidine for polar organic solvents, such as methanol. Its intermediate polarity likely promotes more efficient interactions with methanol compared to highly polar solvents, such as water.

The negative effect of prolonged extraction time is evident when comparing experiments 2 and 6 in the CCRD. This result suggests that longer extraction times may promote degradation of the target compound. A similar effect has been reported for the extraction of bioactive alkaloids from *Stephania tetrandra*,^37^ in which excessive heat compromised compound integrity. Although ultrasound-assisted extraction is efficient for recovering bioactive substances, the cavitation phenomenon can generate localized heat. When applied for extended periods, this process may result in thermal or oxidative degradation of sensitive alkaloids.^22,38^ In the present study, degradation was not directly confirmed, but future work should employ analytical techniques such as LC-MS or additional NMR analyses to identify potential degradation products.

The coefficient of determination (R^2^) value for eq 1 was 0.728. The statistical significance of the model was assessed through analysis of variance (ANOVA), as summarized in Table 3. The regression was significant, with a calculated F-value of 11.6, which was much higher than the tabled F-value (*p* < 0.05). In addition, the lack-of-fit test was not significant (*p* > 0.05), confirming that the model provided a good fit to the experimental data.

**Table 3:** Analysis of Variance (ANOVA) Results for the Regression Model

Variation Source	S.S^a^	D.F^a^	M.S^a^	F-value	p-value
Regression	283.9	3	94.6	11.6	0.0005
Residuals	106.0	13	8.2		
Lack of Fit	104.8	11	9.5	16.9	0.05722
Pure Error	1.1	2	0.6		
Total	389.9	16			

a S.S = Sum of Squares; D.F = Degrees of Freedom; M.S = Mean Square.

### Determination of Optimal Extraction Conditions

Figure 2 shows the response surface and contour plots generated from eq 1. In Figure 2A, the maximum (−)-stepholidine concentration is associated with higher methanol percentages (% MeOH), while the plant-to-solvent ratio has a less pronounced effect. In Figure 2B, shorter extraction times combined with lower plant-to-solvent ratios lead to higher (−)-stepholidine concentrations. In Figure 2C, maximum levels are reached when the methanol percentage (% MeOH) is high and extraction time is reduced. Based on these results, the maximum (−)-stepholidine extraction is predicted to occur at a plant-to-solvent ratio of 1:10, 100% MeOH, and an extraction time of 20 min as the optimal condition.

**Figure 2:**
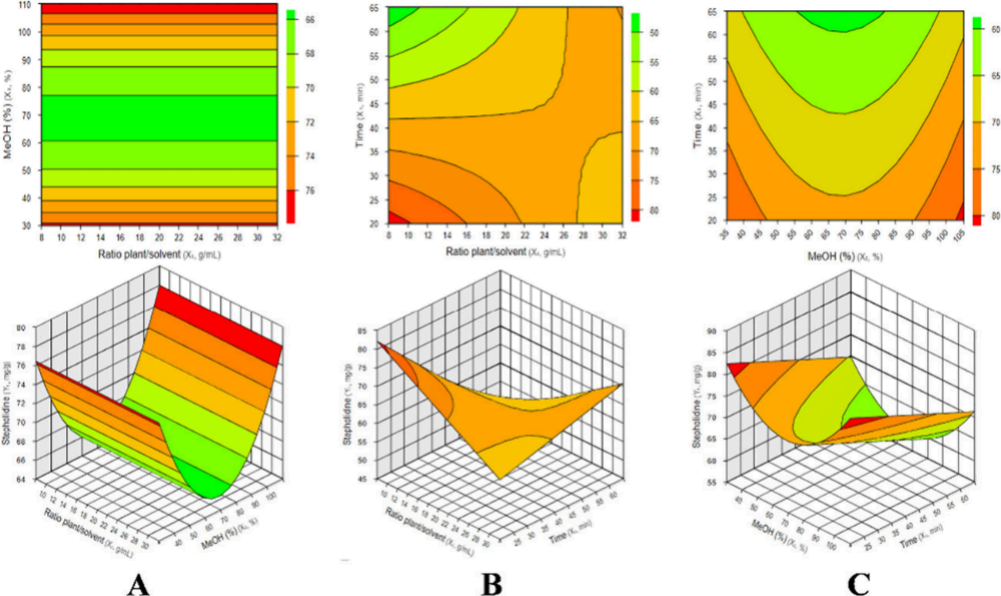
Response surface and contour plots illustrating the effect of independent variables on (−)-stepholidine extraction. (A) Effect of the plant-to-solvent ratio and methanol concentration: the maximum concentration is observed at higher methanol percentages (% MeOH), while the plant-to-solvent ratio has a lesser influence. (B) Effect of the plant-to-solvent ratio and extraction time: higher yields are obtained with shorter extraction times and lower plant-to-solvent ratios. (C) Effect of the methanol concentration and extraction time: maximum (−)-stepholidine levels are reached with high methanol percentages (% MeOH) and reduced extraction times.

To validate the model, the optimized conditions were tested experimentally in triplicate. The extraction yield obtained was 82.8 ± 1.3 mg/g, determined using the PULCON method. This represents an 8.8% increase compared to the best result achieved under the initial, nonoptimized conditions (76.5 ± 2.3 mg/g; Table 1). Furthermore, the experimental yield closely matched the predicted value (81.2 mg/g), corresponding to a prediction accuracy of 98.0%. The close agreement between the predicted and experimental results demonstrates the effectiveness of the statistical model in accurately identifying the optimal extraction conditions. The reliability of the model is supported by the repeatability of the experimental results, which were conducted in triplicate, and the robustness of the parameters used in the modeling.

A previous study employing a statistical mixture design for the extraction of isoquinoline-derived alkaloids from *Unonopsis duckei*, a genus taxonomically related to *Onychopetalum*, demonstrated the superiority of methanol-based solvents. Enhanced extraction yields were reported for anonaine, nornuciferine, glaziovine, norglaucine, and glaucine, with concentrations ranging from 6.79 to 131 μg/g of dried leaves.^35^ In another study, 15 combinations of deep eutectic solvents (DESs) were compared with conventional solvents (methanol, 95% ethanol, and water) for the extraction of the isoquinoline-derived alkaloids fangchinoline and tetrandrine from *Stephania tetrandra*. DESs are green solvents formed by mixing two or more components, typically a hydrogen bond donor and a hydrogen bond acceptor, which interact to form a eutectic mixture. The choice and proportion of components allow the adjustment of the solvent’s physicochemical properties, making it adaptable to specific extraction procedures.^39^ Using RSM, the optimal conditions were defined as an extraction temperature of 52 °C, an extraction time of 82 min, 23% (v/v) DES-water content, and a liquid-to-solid ratio of 23 mL/g. Although DES-based extractions yielded 2.2 times higher concentrations than methanol, the latter still outperformed 95% ethanol and water.^37^ From a green chemistry perspective, it is important to note that although methanol proved to be the most efficient solvent for (−)-stepholidine extraction, it is less environmentally friendly compared to ethanol/water mixtures or deep eutectic solvents (DESs). Ethanol, in particular, represents a safer and renewable alternative, with demonstrated applicability in the extraction of isoquinoline alkaloids in other studies. In future work, it would be valuable to assess the performance of these greener solvents for *Onychopetalum* extracts. Moreover, for processes employing methanol, solvent recovery through rotary evaporation or vacuum distillation offers a feasible route to reduce environmental impact and align the method with sustainable extraction practices.

Similarly, UAE was optimized for the extraction of isoquinoline-derived alkaloids from *Stephania cambodica*, including tetrahydropalmatine, palmatine, and roemerine. The optimal conditions were 52% ethanol, a 9 min extraction time, and a liquid-to-solid ratio of 26.6:1 mL/g. This study highlighted the advantages of UAE, such as minimal plant material usage, shorter processing times, and low operational temperatures.^40^ The UAE approach was further applied to isoquinoline-derived alkaloids from the pulp and byproducts of *Annona muricata* (soursop) using RSM. Ultrasound amplitude (40–100%), time (5–15 min), and pulse cycles (0.4–1 s) were assessed. UAE proved significantly more efficient than conventional maceration, although the complexity of the plant matrix was identified as a key factor affecting extraction efficiency.^41^ Beyond isoquinoline alkaloids, UAE was employed for the extraction of major capsaicinoids from hot peppers, including nordihydrocapsaicin, capsaicin, dihydrocapsaicin, homocapsaicin, and homodihydrocapsaicin. Among the four tested solvents (acetonitrile, methanol, ethanol, and water), methanol at 50 °C for 10 min yielded the best results. This study emphasized the importance of solvent choice, along with other extraction parameters, such as sample mass and solvent volume.^42^

Beyond laboratory-scale optimization, it is worth noting that UAE has strong potential for industrial scale-up. Larger ultrasonic reactors and flow-through systems are already employed in food and pharmaceutical processing, and similar strategies could be adapted for the recovery of isoquinoline alkaloids. Moreover, solvent recovery systems, such as rotary evaporators coupled with condensers or industrial distillation units, allow efficient methanol recycling, reducing both operational costs and environmental impact. These aspects reinforce the practical feasibility of applying the optimized conditions described here to large-scale extraction processes.

## Conclusions

The findings of this study demonstrate that the use of 100% methanol, a 1:10 plant-to-solvent ratio, and a short extraction time of 20 min constitutes an effective and reproducible strategy for maximizing (−)-stepholidine extraction. The excellent agreement between the predicted and experimental values confirms the robustness of the statistical model and its practical applicability. Additionally, comparisons with previous studies highlights methanol as a consistently effective solvent for extracting isoquinoline-derived alkaloids across various plant species and extraction systems. It should be noted that the RSM model developed in this study is specific to methanol as the solvent. Changing the solvent could alter the extraction efficiency due to differences in solubility and interactions with (−)-stepholidine. Therefore, the model should be revalidated or rebuilt when a different solvent is used. These results reinforce the relevance of optimizing solvent composition and extraction time, while also validating the role of response surface methodology as a powerful tool in natural product extraction optimization. Considering the relevance of (−)-stepholidine to the CNS, it is recommended that future work correlates the optimized extraction yields with biological activity assays. In addition, from a practical standpoint, the optimized UAE method shows potential for scale-up to large-scale ultrasonic extraction systems. The use of solvent recovery technologies would further enhance the sustainability and economic viability of methanol-based extraction processes, facilitating future industrial applications. By highlighting efficient extraction strategies, the findings contribute to the valorization of Amazonian biodiversity, emphasizing *O. amazonicum* as a promising source of (−)-stepholidine and providing a foundation for future studies on other isoquinoline-derived alkaloids, supporting the development of phytopharmaceuticals.

## Materials and Methods

### General Experimental Procedures

An analytical knife micromill, model Q298A (Quimis, Diadema, SP, Brazil), equipped with a stainless-steel chamber and blades, operating at 17,000 rpm, was used to grind the samples. An ultrasonic bath, model Q335D (Quimis, Diadema, SP, Brazil), operating at 50 kHz with an ultrasonic power of 135 W, was used in the extraction process. NMR spectroscopy analyses were performed using a Bruker Avance III HD NMR spectrometer (Bruker, Billerica, Massachusetts, USA), operating at 11.7 T (500 MHz for ^1^H) and equipped with a 5 mm BBFO Plus SmartProbe with a *Z*-axis gradient. Whatman grade 1 filter paper (Sigma-Aldrich, St. Louis, MO, USA) was used for filtration. Methanol used for extraction was HPLC-grade purchased from Tedia, and the water was purified using a Milli-Q system. The DMSO-*d*_6_ used for NMR analyses was purchased from Cambridge Isotope Laboratories Inc. (Tewksbury, Massachusetts, USA).

### Plant Material

The leaves of *O. amazonicum* R. E. Fr. (Annonaceae) were collected at the Adolpho Ducke Forest Reserve, located 26 km along the AM-010 highway in the city of Manaus, Amazonas State, Brazil (coordinates: 2°59′15.9″S, 59°55′35.5″W). The specimen had previously been cataloged during the Flora Project. Access to the genetic heritage was registered in the Sistema Nacional de Gestão do Patrimônio Genético e do Conhecimento Tradicional Associado (SisGen) under the registration code No. AE0F182. A voucher specimen (No. 218341) has been deposited in the herbarium of the Instituto Nacional de Pesquisas da Amazônia (INPA). Immediately after collection, the material was air-dried at ambient temperature (approximately 20 °C) for 20 days and properly stored.

### Ultrasound-Assisted Extraction (UAE)

The dried leaves were ground using an analytical knife micromill for 60 s per sample. All samples were processed under identical conditions to ensure the homogeneity of the powdered material prior to extraction. The powdered leaves were transferred to a glass container and mixed with pure methanol or methanol:water solutions, as indicated in Table 1. The extraction was performed in an ultrasonic bath operating in continuous mode at ambient temperature (approximately 20 °C), without the application of external heat. All procedures involving methanol were conducted using appropriate safety measures. After extraction, the samples were filtered using Whatman grade 1 filter paper to remove solid leaf residues. The filtered extracts were then dried in a desiccator at room temperature until complete solvent evaporation. Methanol recovery or recycling was not performed in this study. The dried extracts were transferred to vials and stored in a freezer at – 20 °C until they were prepared for quantitative ^1^H qNMR analysis.

### Extraction Optimization by Design of Experiments

The CCRD (2^3^) based on RSM was applied to evaluate the influence of the plant-to-solvent ratio (g/mL, X_1_), methanol concentration (%, X_2_), and extraction time (min, X_3_) on the UAE of (−)-stepholidine (Y) present in the extract of *O. amazonicum* leaves (Table 2). To avoid thermal degradation and ensure effective extraction, the procedure was carried out under controlled ambient conditions, maintaining a constant temperature and avoiding the use of additional heating.

The experiments were organized according to the three levels evaluated by the CCRD and are presented in Table 1, totaling 17 experimental combinations, including three replicates at the central point.

### Quantitative ^1^H NMR (^1^H qNMR) Analysis Based on PULCON Method

For quantitative analysis by ^1^H NMR, an amount of 20.0 mg (n = 3) of the crude extract was dissolved in 600 μL of DMSO-*d*_6_ and transferred to a 5 mm NMR tube. The zg pulse sequence was used, with the following acquisition parameters: time domain (TD) data points of 65k, spectral width (SW) of 10 kHz, acquisition time (AQ) of 3.27 s, receiver gain (RG) of 32, number of scans (NS) equal to 8, dummy scans of 2, FID resolution of 0.30 Hz and central frequency (O1) set to 3088.30 Hz. The P1 value was automatically calculated for each sample using the *pulsecal sn* command. The D1 value was determined for the signal at δ 6.60 (H-12, d, 8.0 Hz) using eq 2. The longitudinal relaxation time (T1) was measured using the inversion–recovery (*t1ir*) pulse sequence, and the highest T1 value (8.84 s) was used to determine the D1 value for sample acquisition.

2 D1=7×T1−AQ

Dimethyl terephthalate (DMT), a Certified Reference Material (CRM), was provided by the Chemical and Thermal Metrology Division (Inmetro, Rio de Janeiro, Brazil) under the reference number DIMCI1507/2019 (certified purity: 999.88 ± 0.060%). This standard was prepared in triplicate (n = 3) at a concentration of 20.12 mM in DMSO-*d*_6_ (D, 99.9%) with TMS (0.05% v/v) as an internal standard reference (0.00 ppm) and was used as an external standard for quantification via the PULCON method. For the quantitative ^1^H NMR spectrum, the 90° pulse (P1) and the D1 value of DMT were determined for the signal at δ 8.10 (s, 4H). P1 (10.65 μs) was measured using the 90° pulse experiment (*zg*), while the D1 value (16.62 s) was estimated using eq 2, with the acquisition time (AQ) set to 1.64 s. Except for the P1 and D1 parameters, the same acquisition settings used for the quantitative spectra of the extracts were applied to the acquisition of DMT.

Phase and baseline corrections of the spectra were performed manually using TopSpin 3.6.3 software. The chemical shift (in ppm) of the ^1^H NMR spectra was referenced to the solvent, and the coupling constants (J) were recorded in Hz. HSQC and HMBC NMR experiments were also performed. The signal integration of proton H-11 of (−)-stepholidine at δ_H_ 6.76 (*d*, 8.0 Hz)^1^ was performed manually, and the quantification of (−)-stepholidine using the PULCON method was carried out with the ERETIC2 (Electronic REference To access *In vivo* Concentrations) tool in TopSpin 3.6.3 software^43,44^ and the results were expressed as the mean ± standard deviation of the yields obtained (Table 1).

### Statistical Analysis

Statistical calculations during the optimization phase, including model fitting, significance of coefficients (*p* < 0.05), and analysis of variance (ANOVA), were performed using Protimiza Experimental Design software (Protimiza Experimental Design, Brazil). The quantification of (−)-stepholidine by ^1^H qNMR was performed in triplicate (n = 3) for each extract, and the results are expressed as the mean ± standard deviation (SD). The normality of the replicate data was verified using the Shapiro–Wilk test conducted in Minitab 18 (Minitab Inc., USA).

## Supplementary Material



## Data Availability

The authors confirm that the data supporting the findings of this study are available within the article and in the Supporting Information. Further data may be obtained from the corresponding author upon reasonable request.

## Notes

HSQC and HMBC NMR spectra confirming the presence of characteristic signals of (−)-stepholidine in the samples and verifying the absence of signal overlap (PDF)
