# Supplementary material for: Optimization of Ultrasound-Assisted Extraction of (−)-Stepholidine from Onychopetalum amazonicum Leaves Using Response Surface Methodology
Source: ACS Omega. 2025 Nov 11;10(46):55652–9. doi: 10.1021/acsomega.5c06822 (PMC12658633; doi:10.1021/acsomega.5c06822)
Supplement: Supplementary file 1 [file ao5c06822_si_001.pdf]

SUPPORTING INFORMATION

Optimization of Ultrasound-Assisted Extraction of (-)-Stepholidine from *Onychopetalum amazonicum* Leaves Using Response Surface Methodology

Bruna Ribeiro de Lima[1*], Kidney de Oliveira Gomes Neves[2], Lucas Apolinário Chibli[3], Ana Paula Alfaia Castro[2], Rebeca dos Santos França[2], Giovana Anceski Bataglion[1], Marcos Batista Machado[1,2], Afonso Duarte Leão de Souza[1,2], Hector Henrique Ferreira Koolen[4], Maria Lúcia Belém Pinheiro[2], Felipe Moura Araújo da Silva[2,5*]

[1]Departamento de Química, Universidade Federal do Amazonas (UFAM), 69080-900, Manaus - AM, Brazil

[2]Centro de Apoio Multidisciplinar, Universidade Federal do Amazonas (UFAM), 69080-900, Manaus - AM, Brazil

[3]Departamento de Pesquisa e Desenvolvimento de Extratos Botânicos e Desidratados, Duas Rodas, 89251-901, Jaraguá do Sul, SC, Brazil.

[4]Escola Superior de Ciências da Saúde, Universidade do Estado do Amazonas, (UEA), 690065-130, Manaus-AM, Brazil

[5]Coordenação de Tecnologia e Inovação (COTEI), Instituto Nacional de Pesquisas da Amazônia, 69067-375, Manaus-AM, Brazil

[*]Corresponding Author:

Bruna Ribeiro de Lima, E-mail address: bruna.rlima09@gmail.com

Felipe Moura A. da Silva, E-mail address: felipemourams@gmail.com

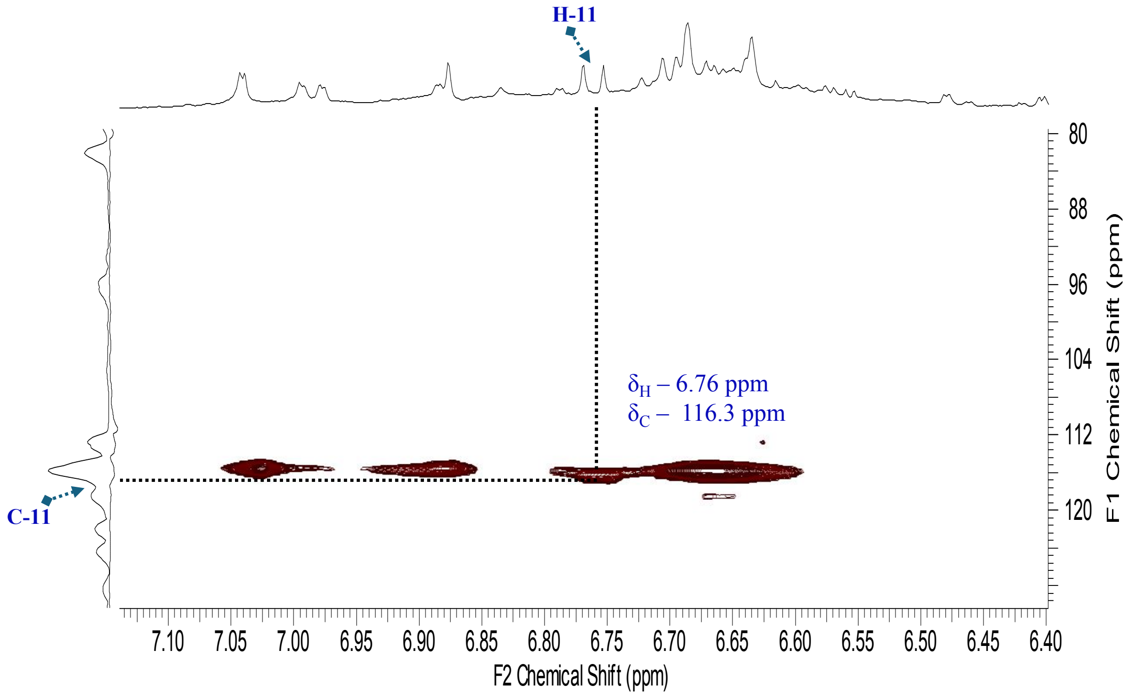

Figure S1. HSQC spectrum of the methanol extract from *O. amazonicum* leaves, highlighting the one-bond correlation between the proton at H-11 and its corresponding carbon.

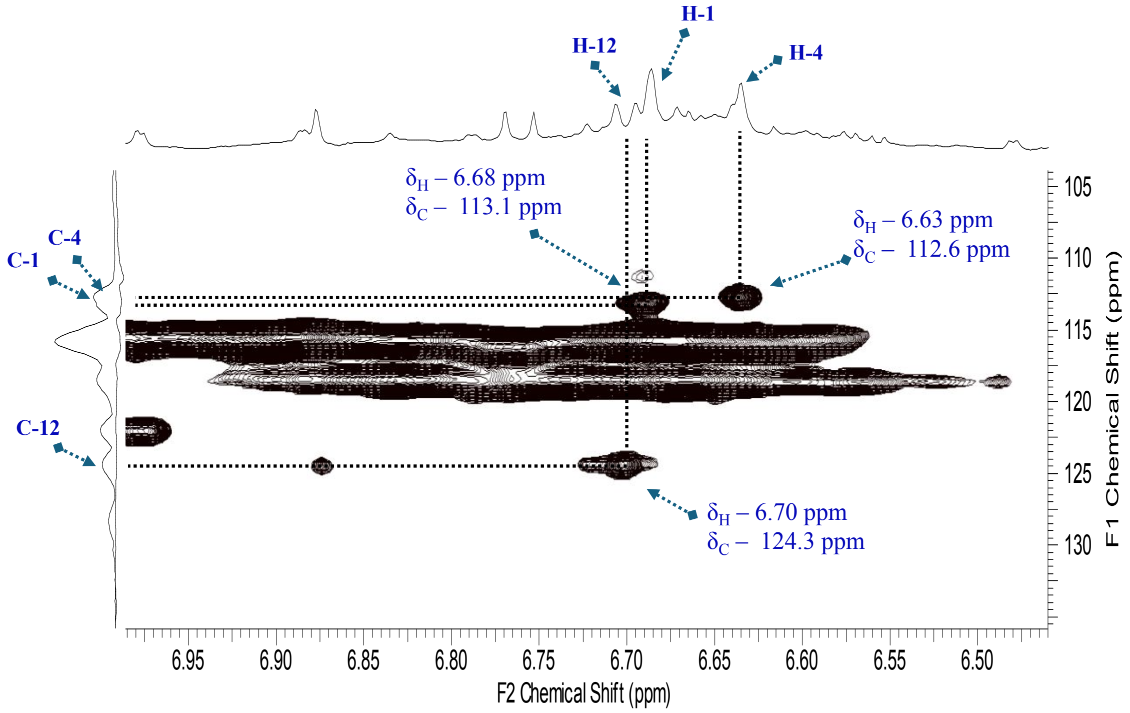

Figure S2. HSQC spectrum of the methanol extract from *O. amazonicum* leaves, highlighting the one-bond correlation between the protons at H-1, H-4, and H-12 and their corresponding carbons.

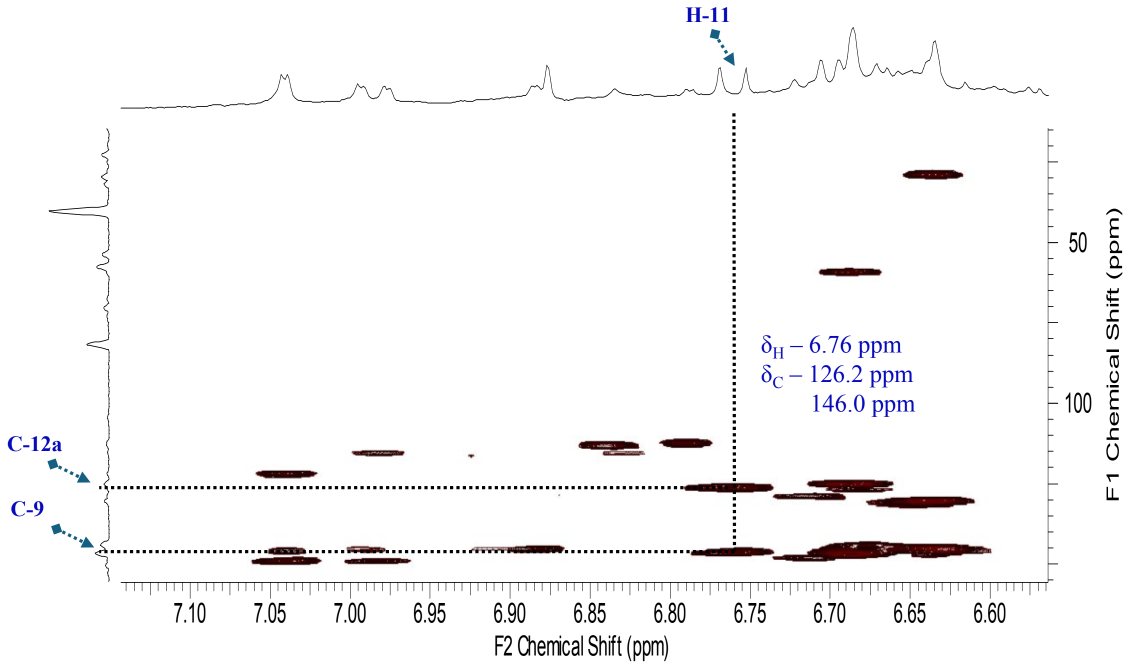

Figure S3. HMBC spectrum of the methanol extract from *O. amazonicum* leaves, highlighting the long-range correlation between the proton at H-11 and key carbons.

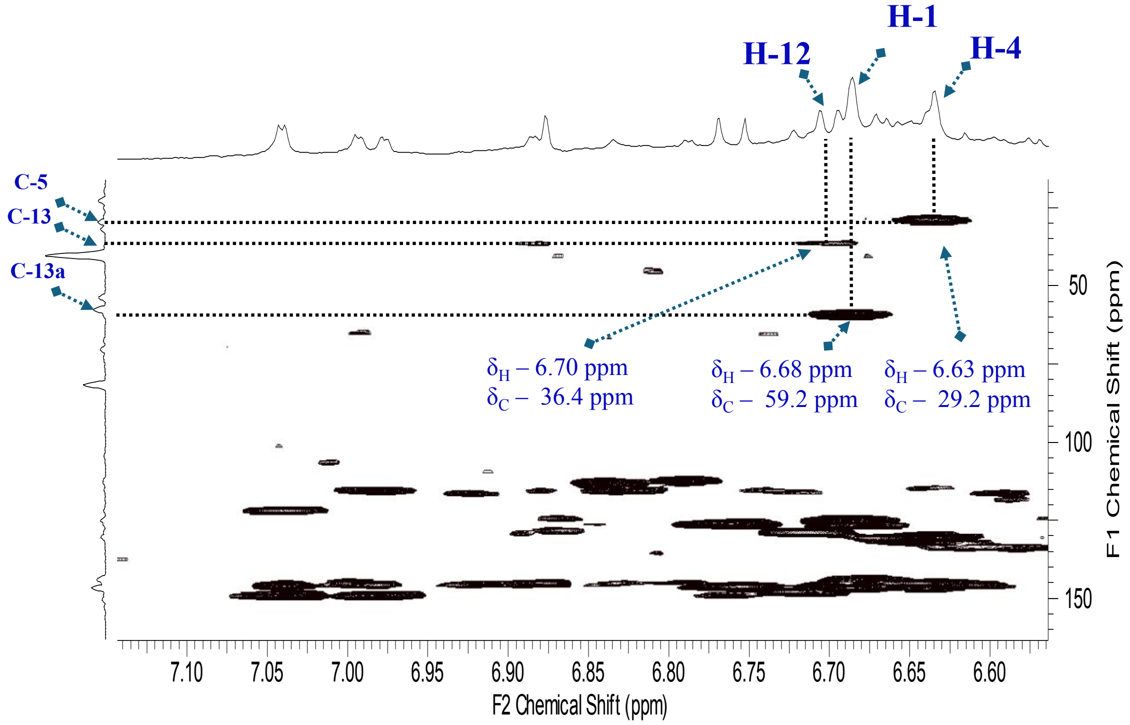

Figure S4. HMBC spectrum of the methanol extract from *O. amazonicum* leaves, highlighting the long-range correlation between the protons at H-1, H-4, and H-12 and key carbons.
